# Case report: First report of potentially zoonotic *Gongylonema pulchrum* in a free-living roe deer (*Capreolus capreolus*) in Slovenia

**DOI:** 10.3389/fvets.2024.1444614

**Published:** 2024-07-26

**Authors:** Petra Bandelj, Diana Žele Vengušt, Gorazd Vengušt, Darja Kušar

**Affiliations:** ^1^Institute of Microbiology and Parasitology, Veterinary Faculty, University of Ljubljana, Ljubljana, Slovenia; ^2^Institute of Pathology, Wild Animals, Fish, and Bees, Veterinary Faculty, University of Ljubljana, Ljubljana, Slovenia

**Keywords:** *Gongylonema pulchrum*, nematode, zoonosis, roe deer (*Capreolus capreolus*), oesophagus, PCR, sequencing

## Abstract

Adult female and male *Gongylonema* nematodes were found in the oesophagus of a free-living roe deer (*Capreolus capreolus*) in Slovenia during passive health surveillance of wildlife. The genus *Gongylonema* was determined by light microscopy based on the genus-specific cuticular bosses in the anterior part of the parasite. Molecular methods were used to confirm the species *Gongylonema pulchrum*, which has zoonotic potential. Although *Gongylonema* species are considered common and distributed worldwide, this is the first report of *G. pulchrum* in an animal on the territory of Slovenia and the first molecular report in a roe deer worldwide. The parasite is likely to be underdiagnosed, misdiagnosed or goes unnoticed as the animals show little or no clinical signs and minor pathological lesions. Slaughterhouse workers, hunters and veterinarians should be aware of this elusive parasite. Examination and evisceration of the upper digestive tract of animals should therefore be carried out more carefully.

## 1 Introduction

*Gongylonema* sp. is a spirurid nematode that forms zigzag patterns in the submucosa of the upper digestive tract of domestic and wild mammals, birds and sometimes humans (1–6). Its main definitive hosts are ruminants, and its global prevalence has been described as common (7–10). In free-living wild herbivores, it has been found in roe deer (*Capreolus capreolus*) (11), European fallow deer (*Dama dama*) (12), bison (*Bison bison*) (13), white-tailed deer (*Odocoileus virginianus*) (14), spotted deer (*Axis axis*), sambar (*Rusa unicolor*), mouse deer (*Tragulus meminna*), nilgai (*Boselaphus tragocamelus*), serow (*Capricornis sumatraensis*), giraffe (*Giraffa camelopardalis*) (15), wild mouflon (*Ovis aries musimon*), sika deer (*Cervus nippon*), feral alien Reeves’s muntjacs (*Muntiacus reevesi*) and water buffalo (*Bubalus bubalis*) (6, 9, 16, 17). The parasite was also found in other game species, such as red fox (*Vulpes vulpes*) and wild boar (*Sus scrofa*) (17). With an estimated population of 10 million animals, the roe deer is the most common and widely distributed deer species in Europe (18). In Slovenia, about 80% of the country’s territory serves as permanent habitat for roe deer, which emphasizes their large presence (19). It is therefore not surprising that the roe deer is one of the most important game species in the country (20).

*Gongylonema* species have an indirect life cycle, in which the intermediate hosts are coprophagous beetles (families *Scarabaeidae*, *Tenebrionidae*, *Hydrophilidae* and *Histeridae*) and some cockroaches (*Blattella* spp.) (21–23). Definitive hosts can become infested by ingesting infested insects or through contaminated food or water (24). Infestation with *Gongylonema* sp. in ruminants usually has no effect on animal health, apart from rare reports of mild to moderate local inflammation with signs of discomfort and irritation in the oesophagus (7, 10). Humans act as accidental hosts, with patients most commonly reporting an intermittent, migratory, worm-like sensations in the upper oesophagus and oral cavity (22, 25–27). An association between *Gongylonema pulchrum* infestation and squamous cell carcinoma was hypothesized in a 17-year-old female ruffed lemur (*Lemur macaco* subsp. *variegatus*) and a 59-year-old man (28, 29). In Slovenia, only one case of autochthonous infestation with *G. pulchrum* in a human was documented in 2019 (22). Although *Gongylonema* sp. is recognized as a parasite of ruminants in Slovenia (30), there are no studies or reports of infestation of animals with this parasite to support this statement.

The aim of this paper is to report the presence of *Gongylonema* sp. in the oesophagus of a free-living roe deer (*C. capreolus*) in Slovenia and its molecular identification as *G. pulchrum* using PCR and Sanger sequencing of the obtained PCR amplicons.

## 2 Case description

**Figure 1:**
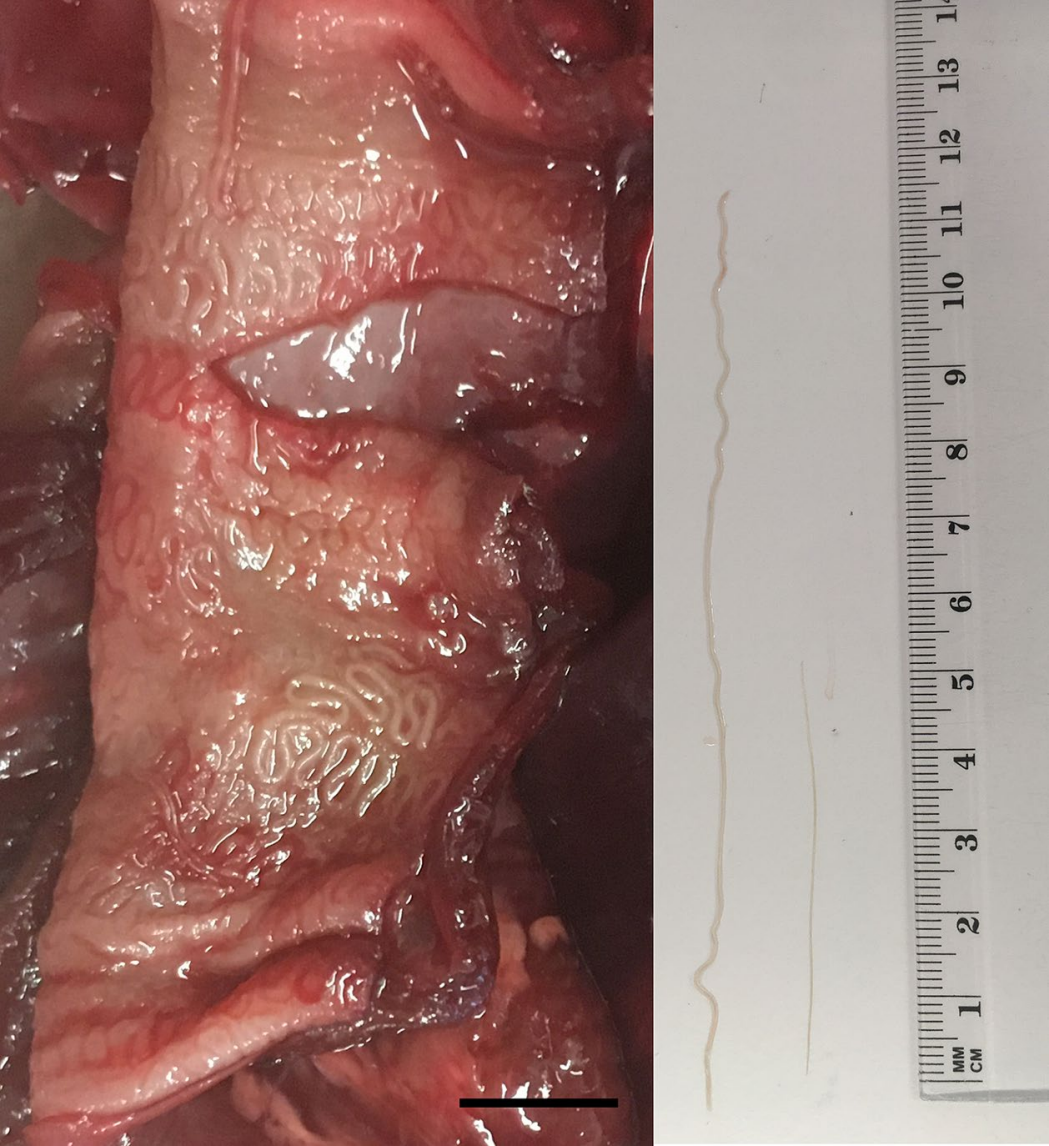
Serpentine-like structures in the subserosa of the oesophagus of a juvenile female roe deer (left); the scale bar represents 1 cm. From the subserosa, white thread-like worms were collected (right); the male (shorter nematode) was approximately 5 cm and the female (longer nematode) 12 cm long.

**Figure 2:**
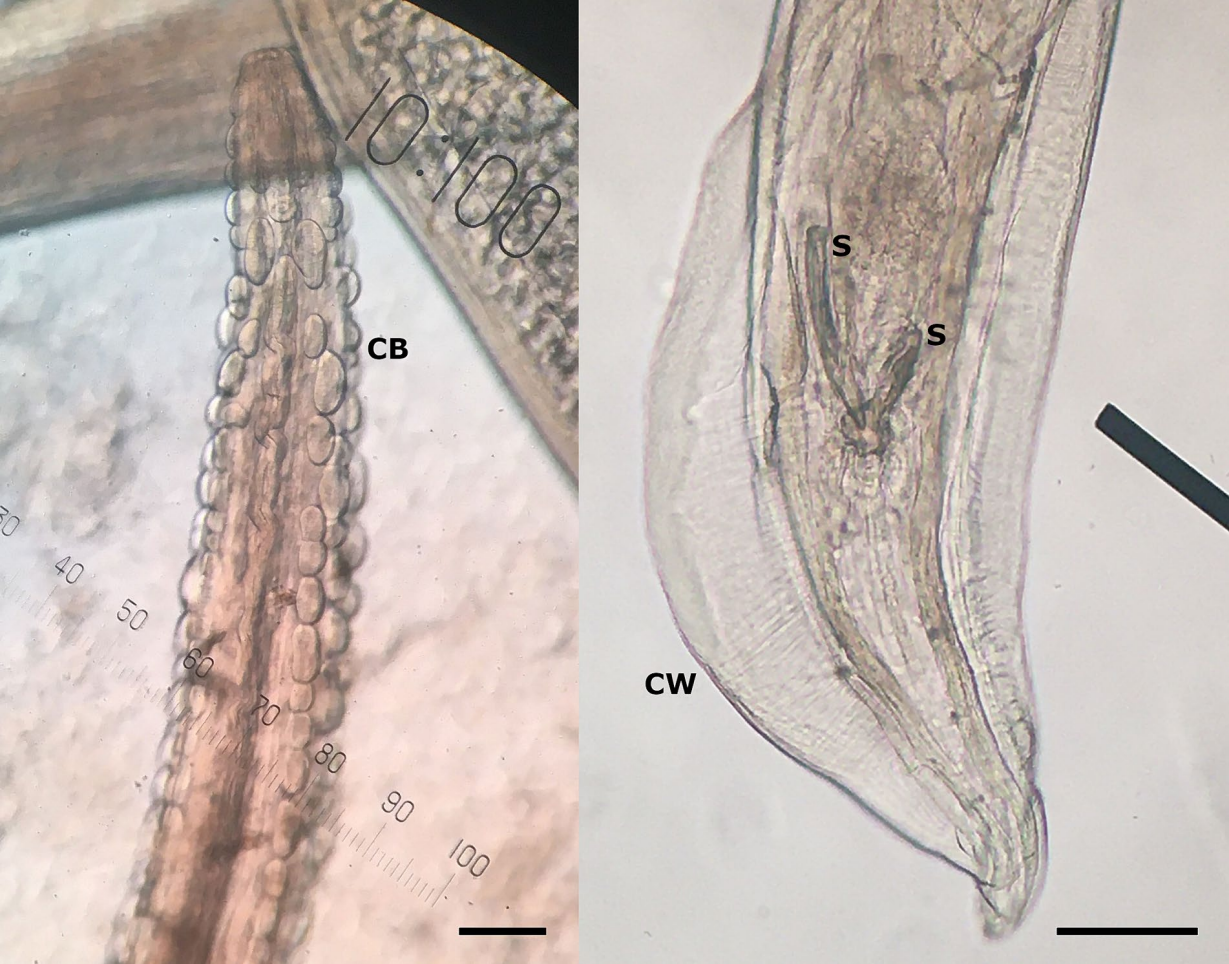
Cuticular bosses (CB) on the anterior part of the nematode, typical for genus *Gongylonema* sp. (left), and posterior part of the male *Gongylonema pulchrum* with caudal wings (CW) and two short spicules (S) of different sizes (right) at 100× magnification. The scale bar represents 100 μm.

In March 2023, the necropsy of a juvenile female roe deer from a hunting ground near the town of Gornji Grad (Lower Styria, Slovenia) was performed at the Veterinary Faculty (Ljubljana, Slovenia) as part of a national passive surveillance programme. The death of the animal followed extensive tissue and organ damage caused by a predator. A detailed parasitological examination of the lungs (for lungworms) and surrounding tissues (heart, oesophagus) unexpectedly revealed serpentine-shape changes in the subserosa of the oesophagus (Figure 1, left). On extraction, the white thread-like worms were 5–12 cm long (Figure 1, right). Under the light microscope, cuticular bosses typical of the genus *Gongylonema* were observed in the anterior part of the parasites (Figure 2, left). A total of ten females and one male were collected. The male was 5 cm long and had asymmetrical caudal wings with two short, differently sized spicules (Figure 2, right), indicating a juvenile male (8). The females were about 12 cm long and had a pronounced uterus filled with embryonated oval eggs.

After morphological examination of the nematodes, molecular methods were used to determine the species; one female nematode was stored in sterile physiological saline solution at −20°C for subsequent molecular analysis. DNA was extracted from the mid-body section of the parasite using the iHelix kit (Institute of Metagenomic and Microbial Technologies, Slovenia; https://www.ihelix.eu/) according to the manufacturer’s instructions. The extraction protocol included bead-beating (45 s at 6400 rpm) three times using a tissue homogenizer (MagNA Lyser Instrument; Roche, Switzerland), combined with enzymatic and heat-induced lysis between mechanical shearings. DNA was eluted to a final volume of 100 μL and stored at −20°C until further analysis. For species determination, PCR and Sanger sequencing were employed, targeting the overlapping segments of the ribosomal RNA (rRNA) genes (rDNA). Twelve universal eukaryotic primer pairs (Supplementary Table S1) were used for PCR amplification as previously described (1, 9, 31); each primer pair was used in a separate PCR reaction. In brief, 25-μl reaction mixtures contained 2.5 μL of DNA, 0.5 U of Platinum Taq DNA Polymerase (Invitrogen by Thermo Fisher Scientific, Waltham, MA, USA), 2.5 mM MgCl_2_ and 1× PCR buffer supplied by the manufacturer, 1 μM of each primer, and 0.25 mM of each dNTP (Applied Biosystems by Thermo Fisher Scientific). Amplification was performed in the VeritiPro Thermal Cycler (Applied Biosystems by Thermo Fisher Scientific) according to the following protocol (applied for all PCR reactions/primer pairs): initial denaturation at 94°C for 3 min, 35 cycles of denaturation at 94°C for 1 min, annealing at 63°C for 1 min, and extension at 72°C for 1 min, and final extension at 72°C for 10 min. The obtained PCR amplicons were analyzed with the QIAxcel capillary electrophoresis system (Qiagen, Germany) using the QIAxcel DNA High Resolution Kit, QX Alignment Marker 15–3,000 bp, QX Size Marker 100–2,500 bp, OM500 separation method and a sample injection time of 10 s according to the manufacturer’s instructions.

Ribosomal PCR amplicons (*n* = 12) were sequenced in both directions (Eurofins Genomics Europe, Germany). The retrieved sequence fragments were imported into Geneious Prime v2022.1.1 (Biomatters, New Zealand) and mapped to a 6,091-bp reference *G. pulchrum* (GeneBank accession no. AB495389.2) to enable reconstruction of a nearly complete *Gongylonema* rDNA region containing also the internal transcribed spacers (ITS) 1 and 2; *G. pulchrum* was selected as suspected according to the origin of the isolate (16). A 6010-bp consensus 18S rDNA - ITS1-5.8S rDNA - ITS2 - 28S rDNA sequence was obtained, which was aligned to three (of 22 available >6,000-bp long *G. pulchrum* sequences of rDNA in GenBank; accessed on 24 April 2024) selected *G. pulchrum* (AB495389.2, AB495394.1, AB495397.1) and one *Gongylonema nepalensis* (LC278392.1) sequence of rDNA. Of note, the retrieved sequence fragments were also mapped to a 6,114-bp reference *G. nepalensis* (LC278392.1) and the consensus sequence obtained was identical to the 6,010-bp consensus after mapping to *G. pulchrum*. The constructed consensus shared most similar single nucleotide polymorphisms (SNPs) and insertions/deletions (indels) to *G. pulchrum* sequences and much less similar to *G. nepalensis*. After the blast search (https://blast.ncbi.nlm.nih.gov/; accessed on April 24, 2024), the consensus sequence was most similar to *G. pulchrum* (100–99.64% identity where query cover was 100%) and less to *G. nepalensis* (97.22–97.07%); lower than 100% query cover (< 93%) was obtained for the *Gongylonema* species *G. aegypti* and *G. neoplasticum*. The results of molecular identification showed that the nematode belonged to *G. pulchrum*. The obtained *G. pulchrum* genomic rDNA sequence, comprising 18S rDNA, ITS1, 5.8S rDNA, ITS2 and 28S rDNA, was submitted to GenBank under the accession number PP594418.

**Figure 3:**
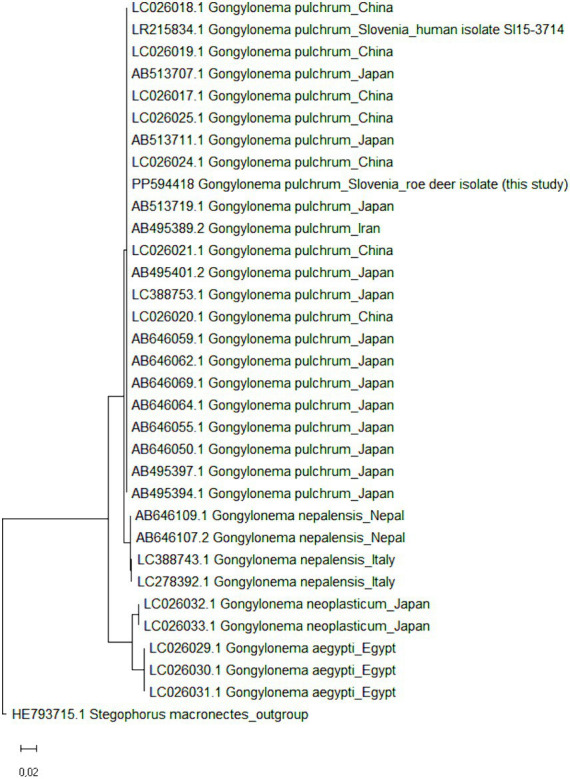
Maximum likelihood phylogenetic tree of >6,000-bp long *Gongylonema* rDNA sequences. GenBank accession numbers are listed in addition to *Gongylonema* species and country of origin. In total, 32 nucleotide sequences of *Gongylonema pulchrum* (*n* = 22 and one from the present study), *Gongylonema nepalensis* (*n* = 4), *Gongylonema neoplasticum* (*n* = 2) and *Gongylonema aegypti* (*n* = 3) were included. The tree is drawn to scale, with branch lengths measured in the number of substitutions per site. *Stegophorus macronectes* rDNA (GenBank accession no. HE793715.1) was used as an outgroup to root the tree.

To confirm the results of molecular identification, all >6,000-bp long rDNA sequences of the genus *Gongylonema* were retrieved from GenBank (accessed on 1 July 2024); a total of 31 rDNA sequences of *G. pulchrum* (*n* = 22 from Japan, China, Iran and Slovenia; the *Gongylonema* isolate from Slovenia was the only one of human origin), *G. nepalensis* (*n* = 4 from Nepal and Italy), *G. neoplasticum* (*n* = 2 from Japan) and *G. aegypti* (*n* = 3 from Egypt) were obtained. The sequences were complemented with the rDNA of *G. pulchrum* obtained in the present study and the phylogenetic tree was constructed in MEGA11 (32) (Figure 3); the maximum likelihood method and Tamura-Nei model were used with default parameters (33). The 6,705-bp long rDNA sequence of *Stegophorus macronectes* (HE793715.1), belonging to the same order (*Rhabditida*) and suborder (*Spirurina*) as *Gongylonema* spp., was used as an outgroup to root the tree. A clear clustering according to *Gongylonema* species was observed, but no sub-species clustering, indicating a high genetic similarity of *G. pulchrum* and the correct identification of the roe deer isolate as *G. pulchrum*.

## 3 Discussion

This is the first molecular report of *G. pulchrum* in a roe deer and the first report of a *Gongylonema* nematode found in an animal in Slovenia. The report also complements the recent human case of *G. pulchrum* reported from Slovenia, which was thought to be an autochthonous infestation (22). The molecular protocols and analyses are presented in detail to facilitate further use in diagnostic laboratories, as many warm-blooded animals are infested with *Gongylonema* nematodes, which are also potential zoonotic agents (1–6).

*Gongylonema* infestation in the oesophagus was discovered at necropsy when a roe deer was found dead in the wild after attack by a predator and examined as part of a national passive health surveillance programme of wildlife in Slovenia. The roe deer population in Slovenia is estimated at around 110,000 animals, with a hunting quota of around 30,000–35,000 animals per year (20, 34). Roe deer are considered the most widespread species of free-living wild ruminants and an important source of game meat in Slovenia (20). In twenty years of passive health surveillance of roe deer, a mortality rate of 26% was recorded for parasitic diseases. In addition to ectoparasites, endoparasites such as *Haemonchus contortus*, *Chabertia ovina* and lung parasites (*Protostrongylidae*, *Dictyocaulus viviparus*) were also detected during the post-mortem examination (35). Until now, not a single *Gongylonema* sp. has been found. The potential infestations in domestic animals and wildlife in Slovenia should be documented as there are no current prevalence reports.

Based on the morphology and the origin of the isolate, *G. pulchrum* was suspected (16). The species was confirmed by sequencing, as the reconstructed 18S rDNA - ITS1-5.8S rDNA - ITS2 - 28S rDNA region was most similar to the rDNA region of *G. pulchrum*. Apart from the isolate obtained in the present study, there are no other *Gongylonema* isolates and corresponding sequences available from ungulates inhabiting Slovenia. Only one *G. pulchrum* rDNA was deposited from our country in 2019 (GenBank accession no. LR215834.1), but it was obtained as part of a report on *G. pulchrum* infestation in a human case (22). In addition, not many studies have generated *Gongylonema* rDNA sequences longer than 6,000 bp, and only two of these sequences (but not belonging to *G. pulchrum*) are from a neighboring country, namely the rDNA of *G. nepalensis* from Italy (GenBank accession nos. LC388743.1 and LC278392.1). The phylogenetic comparison of the rDNA sequences of *G. pulchrum* isolates obtained in Slovenia showed a high genetic similarity between the two sequences, but the same was true for all compared *G. pulchrum* sequences from four geographically distant countries. More sequences (partial rDNA) of *Gongylonema* spp. are available in GenBank, but most of them are shorter and therefore contain much less phylogenetic information (no additional discriminatory power). The high within-species similarity of *Gongylonema* rDNA was also previously described when it was reported that the nucleotide sequences of *G. pulchrum* rDNA were generally well conserved regardless of their host origin (9). We could achieve somewhat greater discriminatory power, if we sequenced the cytochrome c oxidase subunit I (COI) region of mitochondrial DNA (4, 9); the COI sequences of *G. pulchrum* can be further subdivided into several haplotypes (9).

The first and only human case of *G. pulchrum* in Slovenia was self-diagnosed in 2015 and it was later molecularly identified and reported (22). The infestation was described as autochthonous and was most likely due to ingestion of food or water from natural sources thought to be contaminated with the nematode intermediate hosts, dung beetles and cockroaches (26); it was reported that the patient was drinking water from several local springs in the south-eastern part of Slovenia, where there are many grazing areas for livestock (22). Xiaodan et al. (25) reported that the parasite can be overlooked in a patient for more than ten years after infestation. It can also be misdiagnosed as candidiasis, burning mouth syndrome or even a delusional parasitic infestation, as patients report strange crawling sensations in the upper digestive tract (2, 22, 26, 27, 36, 37). The parasite can also contribute to the development of squamous cell carcinoma (28, 29), which can have serious health implications. Therefore, more attention should be paid to *Gongylonema* species, especially *G. pulchrum* with a proven zoonotic potential.

Until the 1980s, *Gongylonema* sp. was frequently reported, with prevalence in domestic ruminants reaching, e.g., 49.7% (276/555) in Iran or up to 96.0% in some regions in Turkey (8, 21). In free-living wild ruminants, a prevalence of 42.8% was reported in 1959 in roe deer from Romania (11, 12) and recently a prevalence of 18.8% (25/133) in European fallow deer (*D. dama*) from Romania (12). In 2013, researchers from Japan reported varying prevalences (from no infestation to a 100% prevalence, depending on sampling location) in sika deer (*C. nippon*) (9). However, over the years, the prevalence in domestic ruminants in the same countries has decreased to, e.g., 4.6% (16/350; Iran) or 0% (0/848, Turkey) in sheep (8, 10) and 16.2% (96/680, Iran), 5.3% (34/638, Japan) or 0.5% (2/380, Turkey) in cattle (7–9). This decrease in prevalence has been attributed to the decline in grazing, the increased use of commercial feeds and the regular use of anthelmintics (8). In Slovenia, *Gongylonema* sp. is mentioned in veterinary parasitology textbooks as a common parasite in the oesophagus of ruminants (30). To our knowledge, there are no published data or reports indicating the prevalence of the parasite. The parasite may be under-reported or under-diagnosed as the clinical signs in animals are usually non-specific, mild and without obvious pathological changes at the site of infestation (7, 10, 25). In this study, the parasite would probably not have been discovered, if the animal had not been attacked by a predator and collected dead by the hunters.

The occurrence of *Gongylonema* sp. in roe deer prompts us to investigate the potential number of cases that may have been overlooked in domestic/captive and wild/free-living animals. This reminds us of the importance of passive health surveillance in wildlife. As regular monitoring activities are associated with high numbers of animals, passive health surveillance of wildlife is particularly important to detect diseases that might otherwise go unnoticed. Slaughterhouse staff, hunters and veterinarians should be educated about this elusive parasite and be vigilant during evisceration or post-mortem examinations. Further studies are essential to reassess the prevalence of *Gongylonema* species in domestic and wild ruminants in Europe and their zoonotic impact.

## Data availability statement

The original contributions presented in the study are publicly available. This data can be found at the National Center for Biotechnology Information (NCBI) using accession number PP594418.

## Ethics statement

Ethical approval was not required for the study involving animals in accordance with the local legislation and institutional requirements as samples were collected post-mortem.

## Author contributions

PB: Conceptualization, Formal analysis, Investigation, Methodology, Resources, Visualization, Writing – original draft, Writing – review & editing. DŽV: Conceptualization, Formal analysis, Investigation, Methodology, Writing – original draft, Writing – review & editing. GV: Conceptualization, Formal analysis, Investigation, Methodology, Writing – original draft, Writing – review & editing. DK: Data curation, Formal analysis, Investigation, Methodology, Software, Validation, Writing – original draft, Writing – review & editing.

## References

[1] Halajian A Eslami A Salehi N Ashrafi-Helan J Sato H. Incidence and genetic characterization of *Gongylonema pulchrum* in cattle slaughtered in Mazandaran province, northern Iran. Iran J Parasitol. (2010) 5:10–8. PMID: 22347239 PMC3279837

[2] Allen JD Esquela-Kerscher A. *Gongylonema pulchrum* infection in a resident of Williamsburg, Virginia, verified by genetic analysis. Am J Trop Med Hyg. (2013) 89:755–7. doi: 10.4269/ajtmh.13-0355, PMID: 23958907 PMC3795108

[3] Esperón F Martín MP Lopes F Orejas P Carrero L Muñoz MJ. *Gongylonema* sp. infection in the scops owl (*Otus scops*). Parasitol Int. (2013) 62:502–4. doi: 10.1016/j.parint.2013.07.005, PMID: 23872068

[4] Setsuda A da N Hasegawa H Behnke JM Rana HB Dhakal IP. Intraspecific and interspecific genetic variation of *Gongylonema pulchrum* and two rodent *Gongylonema* spp. (*G. Aegypti* and *G. Neoplasticum*), with the proposal of *G. nepalensis* n. sp. for the isolate in water buffaloes from Nepal. Parasitol Res. (2016) 115:787–95. doi: 10.1007/s00436-015-4806-3, PMID: 26531300

[5] da Costa CH de Vasconcelos Melo FT Giese EG Santos JND. *Gongylonema* parasites of rodents: a key to species and new data on *Gongylonema neoplasticum*. J Parasitol. (2018) 104:51–9. doi: 10.1645/17-3, PMID: 29135391

[6] Setsuda A Varcasia A Scala A Ozawa S Yokoyama M Torii H. *Gongylonema* infection of wild mammals in Japan and Sardinia (Italy). J Helminthol. (2018) 94:e13. doi: 10.1017/S0022149X18001001, PMID: 30457072

[7] Kheirandish R Radfar MH Sharifi H Mohammadyari N Alidadi S. Prevalence and pathology of *Gongylonema pulchrum* in cattle slaughtered in Rudsar, northern Iran. Sci Parasitol. (2013) 14:37–42.

[8] Gürel T Umur Ş. Prevalence and molecular diagnosis of *Gongylonema pulchrum* in cattle and sheep in the Samsun region. Ankara Univ Vet Fak Derg. (2021) 68:129–35. doi: 10.33988/auvfd.710010

[9] Makouloutou P Setsuda A Yokoyama M Tsuji T Saita E Torii H. Genetic variation of *Gongylonema pulchrum* from wild animals and cattle in Japan based on ribosomal RNA and mitochondrial cytochrome c oxidase subunit I genes. J Helminthol. (2013) 87:326–35. doi: 10.1017/S0022149X12000442 22967753

[10] Eslami A Ashrafihelan J Vahedi N. Study on the prevalence and pathology of *Gongylonema pulchrum* (gullet worm) of sheep from Iran. Global Vet. (2010) 5:45–8.

[11] Stoican E Olteanu G. Contributii la studiul helmintofaunei caprioarei (*Capreolus capreolus*) in R.P.R. (*in Romanian*) *Probl Parazitol Vet*. (1959) 7:38–46.

[12] Popovici DC Marin AM Ionescu O Moraru MMF Kaya DA Imre M. First molecular data of *Gongylonema pulchrum* (Rhabditida: Gongylonematidae) in European fallow deer *Dama dama* from Romania. Pathogens. (2024) 13:175. doi: 10.3390/pathogens13020175, PMID: 38392914 PMC10892342

[13] Demiaszkiewiez AW. Migrations and the introduction of wild ruminants as a source of parasite exchange and emergence of new parasitoses. Ann Parasitol. (2014) 60:25–30. 24930243

[14] Cook TW Ridgeway BT Andrews R Hodge J. Gastro-intestinal helminths in white-tailed deer (*Odocoileus virginianus*) of Illinois. J Wildl Dis. (1979) 15:405–8. doi: 10.7589/0090-3558-15.3.405, PMID: 501845

[15] Chakraborty A. Occurrence and pathology of *Gongylonema* infection in captive wild herbivores. Vet Parasitol. (1994) 52:163–7. doi: 10.1016/0304-4017(94)90047-7, PMID: 8030183

[16] Makouloutou P Rana HB Adhikari B Devkota B Dhakal IP Sato H. A distinct genetic population of *Gongylonema pulchrum* from water buffaloes in Nepal. J Parasitol. (2013) 99:669–76. doi: 10.1645/12-143.1, PMID: 23421498

[17] Varcasia A Scala A Zidda A Cabras PA Gaglio G Tamponi C. First record of *Gongylonema nepalensis* in domestic and wild ruminants in Europe. Vet Parasitol. (2017) 246:11–8. doi: 10.1016/j.vetpar.2017.08.022, PMID: 28969772

[18] Melis C Nilsen EB Panzacchi M Linnell JDC Odden J. Roe deer face competing risks between predators along a gradient in abundance. Ecosphere. (2013) 4:1–12. doi: 10.1890/ES13-00099.1

[19] Adamič M Jerina K. Ungulates and their management in Slovenia In: Apollonio M Andersen R Putman R, editors. European ungulates and their management in the 21st century. Cambridge, United Kingdom: Cambridge University Press (2009). 507–27.

[20] Jerina K Stergar M Videmšek U Kobler A Pokorny B Jelenko I. Spatial distribution, fitness, and population dynamics of ungulates in Slovenia: Studies on the effects of spatially explicite habitat and species-specific factors and predicting future trends (*in Slovene*) [research report]. Ljubljana, Slovenia: University of Ljubljana, Biotechnical Faculty, Department of Forestry and Renewable Forest Resources. (2010). p. 48.

[21] Anwar M Rak H Gyorkos TW. The incidence of Gongylonema pulchrum from cattle in Tehran, Iran. Vet Parasitol. (1979) 5:271–4. doi: 10.1016/0304-4017(79)90016-5

[22] Kramar U Skvarč M Logar M Islamović S Kolenc M Šoba B. First case of human *Gongylonema pulchrum* infection in Slovenia. J Helminthol. (2019) 94:e62. doi: 10.1017/S0022149X19000658, PMID: 31328705

[23] Bravo-Barriga D Martín-Pérez M Lobo JM Parreira R Pérez-Martín JE Frontera E. First detection of *Gongylonema* species in *Geotrupes mutator* in Europe. J Nematol. (2021) 53:e2021–50. doi: 10.21307/jofnem-2021-050, PMID: 34079953 PMC8138951

[24] Wilde H Suankratay C Thongkam C Chaiyabutr N. Human *Gongylonema* infection in Southeast Asia. J Travel Med. (2001) 8:204–6. doi: 10.2310/7060.2001.24242, PMID: 11703903

[25] Xiaodan L Zhensheng W Ying H Hongwei L Jianqiu J Peiru Z. *Gongylonema pulchrum* infection in the human oral cavity: a case report and literature review. Oral Surg Oral Med Oral Pathol Oral Radiol. (2018) 125:e49–53. doi: 10.1016/j.oooo.2017.11.019, PMID: 29329982

[26] Molavi GH Massoud J Gutierrez Y. Human *Gongylonema* infection in Iran. J Helminthol. (2006) 80:425–8. doi: 10.1017/joh2006355 17125553

[27] Ayala MA Yencha MW. *Gongylonema*: a parasitic nematode of the oral cavity. Arch Otolaryngol Head Neck Surg. (2012) 138:1082–4. doi: 10.1001/2013.jamaoto.386 23165385

[28] Bleier T Hetzel U Bauer C Behlert O Burkhardt E. *Gongylonema pulchrum* infection and esophageal squamous cell carcinoma in a Vari (*Lemur macaco variegata*; Kehr 1792). J Zoo Wildl Med. (2005) 36:342–5. doi: 10.1638/04-011.1, PMID: 17323583

[29] Zhou Q Wei Y Zhai H Li S Xu R Li P. Comorbid early esophageal cancer and *Gongylonema pulchrum* infection: a case report. BMC Gastroenterol. (2021) 21:305. doi: 10.1186/s12876-021-01873-8, PMID: 34332527 PMC8325819

[30] Brglez J. Genus *Gongylonema* Molin, 1857 In: Brglez J, editor. Parasitology for veterinarians: Helminthology (*in Slovene*). Ljubljana, Slovenia: University of Ljubljana, Veterinary Faculty (1990). 252–3.

[31] Sato H Suzuki K Aoki M. Nematodes from raccoon dogs (*Nyctereutes procyonoides viverrinus*) introduced recently on Yakushima Island. Japan *J Vet Med Sci*. (2006) 68:693–700. doi: 10.1292/jvms.68.693, PMID: 16891782

[32] Tamura K Stecher G Kumar S. MEGA 11: molecular evolutionary genetics analysis version 11. Mol Biol Evol. (2021) 38:3022–7. doi: 10.1093/molbev/msab120, PMID: 33892491 PMC8233496

[33] Tamura K Nei M. Estimation of the number of nucleotide substitutions in the control region of mitochondrial DNA in humans and chimpanzees. Mol Biol Evol. (1993) 10:512–26. doi: 10.1093/oxfordjournals.molbev.a040023, PMID: 8336541

[34] SiStat (Statistical Office of the Republic of Slovenia). Hunting (number) by game and year (roe deer, 2002–2022). (2022). Available at: https://pxweb.stat.si/SiStatData/pxweb/en/Data/-/1673150S.px/ (Accessed June 5, 2024).

[35] Žele Vengušt D Kuhar U Jerina K Vengušt G. Twenty years of passive disease surveillance of roe deer (*Capreolus capreolus*) in Slovenia. Animals. (2021) 11:407. doi: 10.3390/ani11020407, PMID: 33562662 PMC7915899

[36] Eberhard ML Busillo C. Human *Gongylonema* infection in a resident of new York City. Am J Trop Med Hyg. (1999) 61:51–2. doi: 10.4269/ajtmh.1999.61.51, PMID: 10432055

[37] Haruki K Furuya H Saito S Kamiya S Kagei N. *Gongylonema* infection in man: a first case of gongylonemosis in Japan. Helminthologia. (2005) 42:63–6.

